# Weight Loss in Midlife, Chronic Disease Incidence, and All-Cause Mortality During Extended Follow-Up

**DOI:** 10.1001/jamanetworkopen.2025.11825

**Published:** 2025-05-27

**Authors:** Timo E. Strandberg, Arto Y. Strandberg, Satu Jyväkorpi, Annele Urtamo, Solja T. Nyberg, Philipp Frank, Jaana Pentti, Kaisu H. Pitkälä, Mika Kivimäki

**Affiliations:** 1 University of Helsinki, Helsinki, Finland; 2 Helsinki University Hospital, Helsinki, Finland; 3 University of Oulu, Center for Life Course Health Research, Oulu, Finland; 4 Clinicum, University of Helsinki, Helsinki, Finland; 5 Finnish Institute of Occupational Health, Helsinki, Finland; 6 Brain Sciences, University College London, London, United Kingdom; 7 Department of Public Health, University of Turku, Turku, Finland; 8 Centre for Population Health Research, University of Turku, Turku, Finland

## Abstract

**Importance:**

Few studies have examined long-term health benefits among individuals with sustained weight loss beyond its association with decreased diabetes risk.

**Objective:**

To examine the long-term association of body mass index (BMI; calculated as weight in kilograms divided by height in meters squared) changes during healthy midlife (ages 40-50 years) with later-life morbidity and mortality.

**Design, Setting, and Participants:**

This cohort study analyzed data from 3 cohorts that included repeated height and weight measurements: the Whitehall II study (WHII; baseline, 1985-1988), Helsinki Businessmen Study (HBS; baseline, 1964-1973), and Finnish Public Sector study (FPS; baseline, 2000). Participants were categorized into 4 groups based on their first 2 weight assessments and followed up for morbidity and mortality outcomes. Data analyses were conducted between February 11, 2024, and February 20, 2025.

**Exposures:**

Midlife BMI change was categorized as persistent BMI less than 25, BMI change from 25 or greater to less than 25, BMI change from less than 25 to 25 or greater, and persistent BMI of 25 or greater.

**Main Outcomes and Measures:**

Incident chronic disease, including type 2 diabetes, myocardial infarction, stroke, cancer, asthma, or chronic obstructive pulmonary disease, was assessed in WHII and FPS, and all-cause mortality was assessed in HBS. These outcomes were obtained from linked electronic health records in national health registries.

**Results:**

There were 23 149 participants, including 4118 men and women (median [IQR] age at first visit, 39 [37-42] years; 2968 men [72.1%]) from WHII, 2335 men (median [IQR] age at first visit, 42 [38-45] years) from HBS, and 16 696 men and women (median [IQR] age at first visit, 39 [34-43] years; 13 785 women [82.6%]) from FPS. During a median (IQR) follow-up of 22.8 (16.9-23.3) years, after adjusting for smoking, systolic blood pressure, and serum cholesterol at the first evaluation, WHII participants with weight loss had a decreased risk of developing chronic disease (hazard ratio [HR], 0.52; 95% CI, 0.35-0.78) compared with participants with persistent overweight. This finding was replicated after excluding diabetes from the outcome (HR, 0.58; 95% CI, 0.37-0.90). The corresponding HR in FPS was 0.43 (95% CI, 0.29-0.66) over a median (IQR) follow-up of 12.2 (8.2-12.2) years. In HBS, weight loss was associated with decreased mortality (HR, 0.81; 95% CI, 0.68-0.96) during an extended follow-up (median [IQR], 35 [24-43] years).

**Conclusions and Relevance:**

In this study, conducted when surgical and pharmacological weight-loss interventions were nearly nonexistent, sustained midlife weight loss compared with persistent overweight was associated with a decreased risk of chronic diseases beyond type 2 diabetes and decreased all-cause mortality.

## Key Points

**Question:**

Is sustained, nonsurgical, and nonpharmacological weight reduction in midlife associated with long-term health benefits beyond decreased diabetes risk?

**Findings:**

In this cohort study of 23 149 adults from 3 cohorts with repeated height and weight measurements in 12- to 35-year follow-ups, sustained weight loss from overweight to healthy weight in midlife was associated with decreased risk of chronic diseases, including and excluding type 2 diabetes, and decreased all-cause mortality compared with persistent overweight.

**Meaning:**

This study found that sustained midlife weight reduction achieved without surgical or pharmacological interventions was associated with long-term health benefits beyond its associations with decreased diabetes risk.

## Teaser

This cohort study investigates the association of sustained weight loss in midlife with long-term health benefits.

## Introduction

Metabolic-bariatric surgery and glucagon-like peptide 1 receptor agonist treatment for morbid obesity, both leading to substantial weight loss, are associated with a decreased incidence of major clinical end points, at least within follow-up periods of less than 10 years.^1,2^ Additionally, randomized clinical trials (RCTs) have shown that behavioral weight loss interventions in individuals with overweight and obesity decrease diabetes risk.^3,4,5,6^ However, the long-term association of lifestyle interventions with other major diseases has remained unclear, with mixed findings regarding cardiovascular outcomes and mortality in observational studies and RCTs of weight reduction.^7,8,9,10,11,12,13,14,15,16,17,18,19^ A 2022 meta-analysis^20^ concluded that there is insufficient evidence supporting behavioral weight loss interventions for cardiovascular prevention among individuals with overweight and type 2 diabetes.

Methodological limitations may have contributed to the ongoing uncertainty regarding the effectiveness of nonsurgical and nonpharmacological weight loss interventions. Findings from the Da Qing Diabetes Prevention Study^21^ among Chinese individuals with impaired glucose tolerance suggest that long-term follow-up is essential to confirm the benefits associated with midlife weight reduction. This 6-year lifestyle intervention was associated with decreased cardiovascular and total mortality, but these benefits emerged only after 30 years of follow-up.^21^ To our knowledge, no independent studies have confirmed these findings or replicated them in other ethnic groups.

Although intentional weight reduction is generally beneficial, it can also have adverse effects, including the loss of lean body mass.^22^ Therefore, more research is needed to assess key clinical outcomes, such as morbidity and mortality, particularly in individuals without diabetes, after weight reduction over extended follow-up periods.^18^ To address this, we examined associations between reductions in body mass index (BMI; calculated as weight in kilograms divided by height in meters squared) without surgical or pharmacological treatment during healthy midlife (ages 40-50 years) and long-term incidence of chronic diseases and all-cause mortality.

## Methods

The primary analysis of this cohort study was based on 2 cohort studies analyzed separately: the Whitehall II (WHII) study^23^ and Helsinki Businessmen Study (HBS).^24^ Analyses were replicated in a third cohort, the Finnish Public Sector (FPS) study.^25^ In all 3 cohort studies, participants provided written informed consent prior to their involvement in the study. Ethics approval was obtained from the University College London Hospital Committee on the Ethics of Human Research for WHII, Helsinki University Hospital Ethics Committee for HBS, and Ethical Committee of the Helsinki Uusimaa Hospital District for FPS. Ethics approvals and satisfaction of informed consent requirements extend to this study, which followed the Strengthening the Reporting of Observational Studies in Epidemiology (STROBE) reporting guideline for cohort studies.

### Study Population and Design

**Figure 1.**
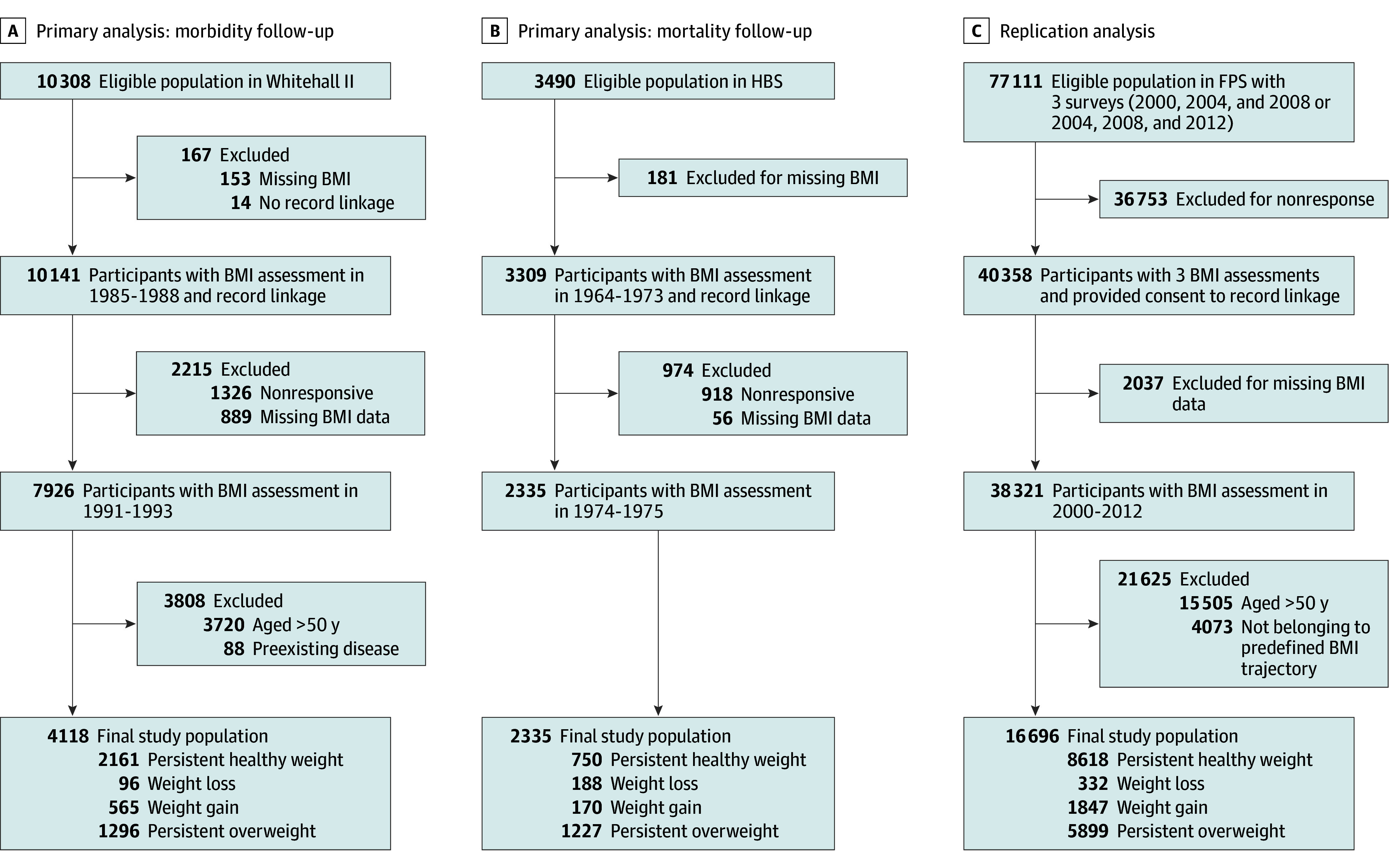
Selection of Study Participants Participant selection is shown for the Whitehall II study, Helsinki Businessmen Study (HBS), and Finnish Public Sector (FPS) study. BMI indicates body mass index.

The WHII cohort consisted of 10 308 British civil servants aged 35 to 55 years at baseline (1985-1988, first evaluation).^23^ Clinical examinations occurred between 1991 and 2013, and participants were linked to electronic health records (EHRs) from National Health Service hospital admissions and death registries. We included 4118 men and women aged 39 to 49 years who were free of chronic disease at the second evaluation (1991-1993) (Figure 1).

In the HBS, 3490 White men, mostly businessmen and executives born between 1919 and 1934, aged 31 to 54 years, and free of chronic diseases, underwent voluntary health checkups from 1964 to 1973 (first evaluation).^24^ Cardiovascular risk factors were assessed using standard methods, and the men received health education on weight control (diet and exercise) and cardiovascular risk reduction. In 1974 to 1975 (second evaluation; age range, 40-55 years), participants were assessed again, including measured body weight. Clinical data from both evaluations were available for 2335 men, who formed the sample for this study.

The FPS is an occupational cohort of 77 111 men and women with BMI assessed 3 times at 4-year intervals using self-reported data from surveys conducted between 2000 and 2013. Participants were linked to EHRs. We included 16 696 men and women who had data from 3 consecutive BMI assessments, were younger than 50 years, had no prevalent chronic disease at the second evaluation, and were successfully linked to EHRs.

In each cohort, we categorized participants into 4 weight groups based on BMI assessments at first and second clinical evaluations: persistent healthy weight (BMI <25), weight loss from overweight to healthy weight (BMI change from ≥25 to <25), weight gain from healthy weight to overweight (BMI change from <25 to ≥25), and persistent overweight (BMI ≥25). Only participants free (or mostly free in the HBS) of the studied diseases at the second evaluation were included, and they were followed up for incident disease and mortality thereafter. This study followed a predefined protocol to synthesize evidence (CRD42022328472).

Measurement of weight and height was conducted at a time when surgical and pharmacological weight-loss interventions were nearly nonexistent. The cause of weight loss was not assessed, but given the age of the participants and lack of diagnosed disease, it was more likely intentional than caused by severe chronic conditions or frailty. In all cohorts, the median age of participants in all weight groups was younger than 50 years at the second evaluation.

### Baseline Assessments

Measurement of weight and height in the first and second evaluation is described in the eMethods in Supplement 1. In WHII and HBS, covariates in the first evaluation were assessed according to standard operating protocols: age, sex, smoking history, systolic and diastolic blood pressure, and total cholesterol. In FPS, no data were available on blood pressure or cholesterol. For this cohort, participants were linked to EHRs of the Finnish Prescription Register and hypertension was identified if the participants had reimbursed prescriptions of antihypertensive medication as defined by World Health Organization (WHO) Anatomical Therapeutic Chemical codes C02, C03, C07, C08, and C09. Additional clinical characteristics of participants were collected, including measured postload glucose in WHII and HBS, self-reported physical activity (high, intermediate, and low) in WHII only, and disease status beyond health records in HBS (eTable 1 in Supplement 1).

### Morbidity and Mortality Follow-Up

In WHII, participants were linked to national registers for hospitalization and vital status during follow-up. Our outcome of interest was any of the following: incident type 2 diabetes (*International Statistical Classification of Diseases and Related Health Problems, Tenth Revision *[*ICD-10*] code E11), nonfatal myocardial infarctions (*ICD-10* codes I21-I22), coronary deaths (*ICD-10* codes I20-I25), stroke (*ICD-10* codes I60, I61, I63, and I64), cancers (*ICD-10* codes C00-C97), asthma (*ICD-10* codes J45-J46), and chronic obstructive pulmonary disease (COPD) exacerbations (*ICD-10* codes J41, J42, J43, and J44). These end points are defined by WHO as being associated with premature mortality.

In HBS, participants were linked to records of the national death registry for all-cause mortality, Statistics Finland, through January 31, 2021. In FPS, participants were linked to mortality (Statistics Finland) and hospital discharge (Finnish Institute for Health and Welfare) registries until December 31, 2018, including the same *ICD-10* codes as in WHII. Additional information on cancers, diabetes, asthma, and COPD was available via record linkage to the Drug Reimbursement Register of the Social Insurance Institution.

### Statistical Analysis

Analyses were performed between February 11, 2024, and February 20, 2025. In the analysis of participant characteristics at the first evaluation, we used the median test and analysis of variance to compare continuous variables and χ^2^-test to compare proportions. After assessing proportionality with Schoenfeld residuals, we used Cox regression to examine associations of weight groups (first and second evaluations) with the risk of morbidity and mortality after the second evaluation. Hazard ratios (HRs) with their 95% CIs were adjusted for age, sex, smoking history, systolic blood pressure, and total cholesterol at the first evaluation in WHII and HBS and for age, sex, and hypertension in FPS. Participants with missing covariate values were excluded given that the proportion of missing data was less than 1% (eTable 2 in Supplement 1).

To assess whether BMI changes after baseline could introduce bias, we included BMI change after the second evaluation as a time-dependent covariate in the model. To examine sex differences, we stratified Cox regression analyses by sex. Given that the association between weight change and disease risk in women may vary by menopausal status, we analyzed these associations separately for diseases with onset before age 50 years and at age 50 years or older.

We used 2-tailed tests and considered 2-sided *P* values < .05 to be significant. SAS statistical software version 9.4 (SAS Institute) was used to analyze data (eAppendix in Supplement 1). In HBS, NCSS statistical software version 2020 (NCSS, LLC) was additionally used.

## Results

Among 23 149 participants in all 3 cohorts, there were 4118 men and women (median [IQR] age at first visit, 39 [37-42] years; 2968 men [72.1%] and 1150 women [27.9%]) in WHII, 2335 men (median [IQR] age at first visit, 42 [38-45] years) in HBS, and 16 696 men and women (median [IQR] age at first visit, 39 [34-43] years; 2911 men [17.4%] and 13 785 women [82.6%]) in FPS. In the WHII cohort, 2161 participants maintained a persistent healthy weight in the first and second evaluation, 96 participants lost weight and transitioned from overweight to healthy weight, 565 participants gained weight and shifted from healthy to overweight, and 1296 participants had persistent overweight (Table 1). In the HBS cohort, the corresponding numbers were 750 participants, 188 participants, 170 participants, and 1227 participants, respectively. While median BMIs calculated from recalled weight at age 25 were within the healthy BMI range in all groups of WHII and HBS, the highest median BMI was observed among participants with persistent overweight already in young adulthood. Participants stayed on their respective BMI trajectories during the follow-up until age 66 years in WHII and age 80 years in HBS, at which point weight loss and weight gain groups began to converge (eFigure 1 in Supplement 1).

**Table 1.** Study Participants at First and Second Evaluations

Characteristic	Whitehall II						HBS					
	Participants, No. (%)					*P* value	Participants, No. (%)					*P* value
	All (n = 4118)	Persistent healthy weight (n = 2161)^a^	Weight loss (n = 96)^a^	Weight gain (n = 565)^a^	Persistent overweight (n = 1296)^a^		All (n = 2335)	Persistent healthy weight (n = 750)^a^	Weight loss (n = 188)^a^	Weight gain (n = 170)^a^	Persistent overweight (n = 1227)^a^	
**First evaluation**												
Sex												
Men	3004 (72.9)	1558 (72.1)	73 (76.0)	410 (72.6)	963 (74.3)	.47	2335 (100)	750 (100)	188 (100)	170 (100)	1227 (100)	>.99
Women	1114 (27.1)	603 (27.9)	23 (24.0)	155 (27.4)	333 (26.7)		NA	NA	NA	NA	NA	
Age, median (IQR), y	39 (37 to 42)	39 (37-41)	39 (37-42)	39 (37-42)	40 (38-42)	.005	42 (38-45)	42 (38-45)	42 (38-45)	41 (38-45)	42 (39-45)	.47
BMI, mean (SD)												
Overall	24.2 (3.2)	22.1 (1.5)	26.2 (2.0)	24.0 (0.8)	27.8 (2.7)	<.001	25.8 (2.7)	23.1 (1.4)	25.9 (0.9)	24.1 (0.9)	27.7 (2.1)	<.001
At age 25 y	22.4 (2.8)	21.1 (1.8)	23.5 (2.8)	22.3 (1.9)	24.6 (3.0)	<.001	22.7 (2.2)	21.7 (1.7)	22.8 (1.8)	22.5 (1.9)	23.4 (2.2)	<.001
Obesity	208 (5.1)	0	2 (2.1)	0	206 (15.9)	<.001	167 (7.2)	0	1 (0.5)	0	166 (13.5)	<.001
Height, mean (SD), cm												
Men	177 (6.6)	177 (6.6)	176 (6.9)	177 (6.5)	176 (6.6)	.001	177 (5.9)	177 (6.1)	177 (5.8)	177 (6.1)	177 (5.7)	.38
Women	163 (6.5)	164 (6.6)	161 (7.1)	162 (6.6)	162 (6.2)	.001	NA	NA	NA	NA	NA	NA
Smoking history												
Current smoker	644 (15.8)	325 (15.2)	18 (19.0)	104 (18.6)	197 (15.3)	.19	NA	NA	NA	NA	NA	NA
Former smoker	1273 (31.2)	621 (29.0)	28 (29.5)	167 (29.8)	457 (35.6)	.001	NA	NA	NA	NA	NA	NA
Current or former smoker	1917 (47.0)	946 (44.1)	46 (48.4)	271 (48.4)	654 (50.9)	.001	1543 (66.1)	482 (62.3)	111 (59.0)	112 (65.9)	838 (68.3)	.047
BP, mean (SD), mm Hg												
Systolic	121.4 (13.3)	119.9 (13.0)	123.9 (15.0)	119.8 (12.9)	124.3 (13.3)	<.001	135 (14.9)	131 (13.4)	135 (13.2)	132 (12.7)	137 (15.8)	<.001
Diastolic	75.5 (9.5)	73.8 (9.4)	76.7 (10.3)	75.2 (9.0)	78.3 (9.3)	<.001	86 (9.7)	83 (8.7)	86 (8.6)	84 (8.5)	87 (10.3)	<.001
Total cholesterol, mean (SD), mg/dL	220.2 (42.5)	213.2 (39.5)	225.9 (42.6)	219.6 (40.5)	231.8 (43.4)	<.001	255 (44.8)	251 (43.1)	261 (47.0)	253 (42.3)	258 (45.6)	.005
**Second evaluation**												
Age, median (IQR), y	45 (42-47)	44 (42-47)	45 (42-47)	45 (43-47)	45 (43-47)	.002	49 (45-52)	49 (45-52)	49 (45-52)	48 (45-51)	49 (46-52)	.21
Years since first evaluation, mean (SD)	5.2 (0.7)	5.2 (0.7)	5.2 (0.7)	5.3 (0.7)	5.2 (0.7)	.003	7.0 (2.3)	7.0 (2.3)	7.0 (2.2)	7.1 (2.4)	7.0 (2.3)	.92
BMI, mean (SD)	25.1 (3.6)	22.6 (1.5)	24.2 (1.0)	26.1 (1.1)	29.0 (3.3)	<.001	25.9 (2.8)	23.2 (1.4)	24.2 (0.6)	26.0 (0.9)	27.9 (2.2)	<.001
Obesity	364 (8.8)	0	0	7 (1.2)	357 (27.6)	<.001	177 (7.6)	0	0	0	177 (14.4)	<.001
Weight change, mean (SD), kg	2.6 (4.6)	1.5 (3.3)	−5.2 (7.0)	6.0 (3.7)	3.6 (5.2)	<.001	0.3 (4.6)	0.1 (3.3)	−5.4 (3.3)	5.9 (4.1)	0.6 (4.6)	<.001
Relative weight change (SD), %	3.7 (6.2)	2.4 (5.1)	−6.5 (7.8)	8.5 (7.8)	4.4 (6.2)	<.001	0.5 (5.7)	0.2 (4.5)	−6.5 (3.7)	8.0 (6.1)	0.7 (5.2)	<.001

Abbreviations: BMI, body mass index (calculated as weight in kilograms divided by height in meters squared); BP, blood pressure; HBS, Helsinki Businessmen Study; NA, not applicable.

SI conversion factors: To convert cholesterol to millimoles per liter, multiply by 0.0259.

^a^ Weight change is between the first and second evaluations. The first and second evaluations were in 1985 to 1988 and 1991 to 1993 in Whitehall II and 1964 to 1973 and 1974 to 1975 in HSB. For the Finnish Public Sector study, see the eMethods in Supplement 1. Healthy weight was defined as a BMI less than 25, and overweight was defined as a BMI of 25 or greater.

In the first 2 examinations, the prevalence of obesity (BMI ≥30) was low in both cohorts (WHII: 208 participants [5.1%] and 364 participants [8.8%]; HBS: 167 participants [7.2%] and 177 participants [7.6%]) (Table 1). While systematic differences in smoking, blood pressure, total cholesterol, and postload glucose between participants with weight loss and those with persistent overweight were small at the first evaluation (Table 1), blood pressure and total cholesterol at the second evaluation were lower in the weight loss group (eTable 3 in Supplement 1). Similarly, based on concentrations in the 2-hour postload glucose measurement in WHII and the 1-hour postload glucose measurement in HBS, the threshold for prediabetes was exceeded by 5 participants (5.4%) vs 130 participants (10.7%) in the groups with weight loss and persistent overweight at the second evaluation in WHII, with corresponding prevalences of 27 participants (14.6%) and 279 participants (22.7%) in HBS. In WHII, the proportion of physical inactivity increased in all weight groups, except participants with weight loss (eTable 3 in Supplement 1).

**Figure 2.**
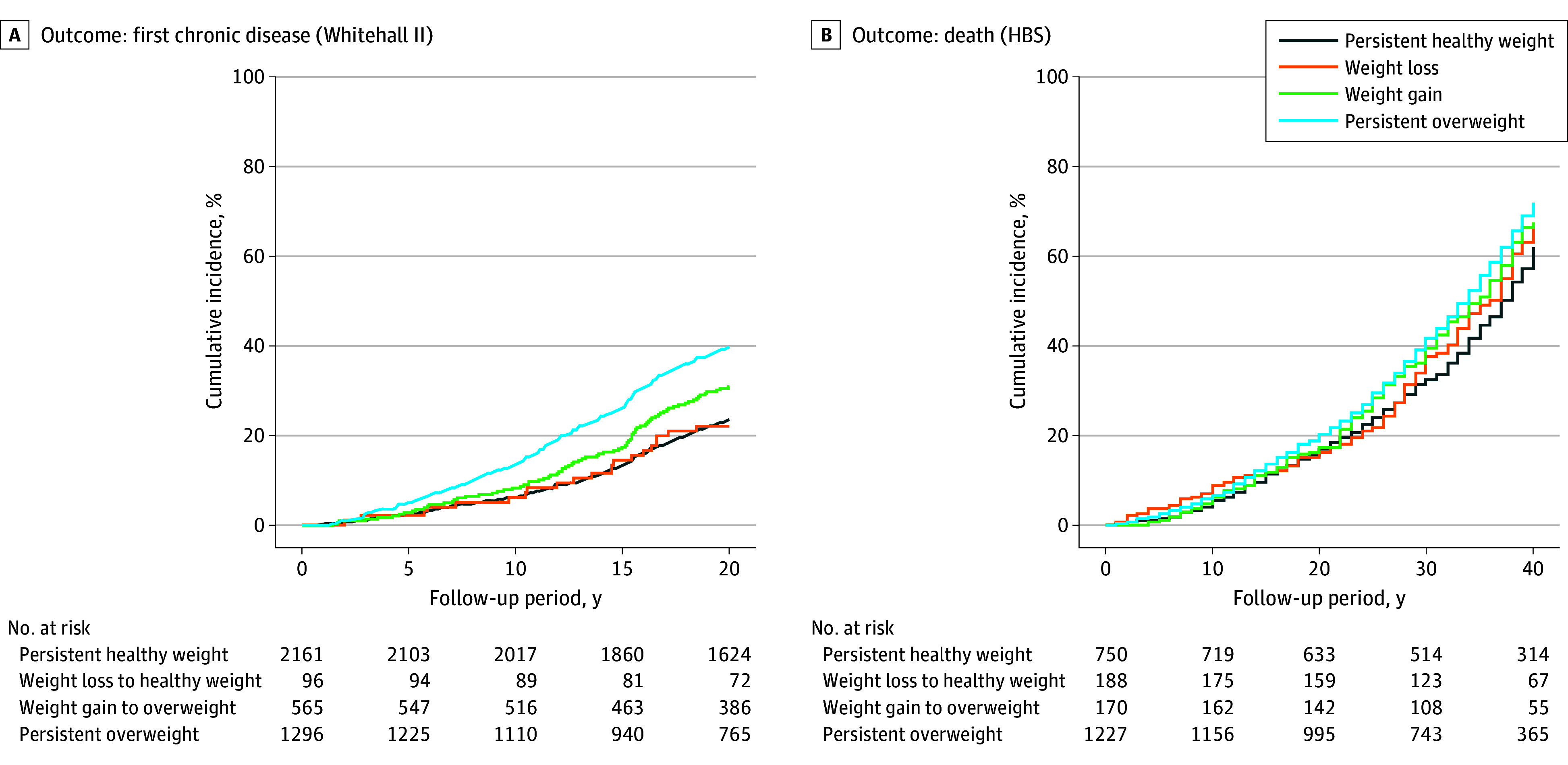
Cumulative Hazard of Incident Chronic Disease and Mortality in Primary Analysis HBS indicates Helsinki Businessmen Study.

By the end of follow-up in WHII, 634 participants (29.3%) with persistent healthy weight, 26 participants (27.1%) with midlife weight loss, 204 participants (36.1%) with weight gain, and 586 participants (45.2%) with persistent overweight developed at least 1 chronic condition. The excess morbidity risk associated with midlife weight gain and persistent overweight was apparent across the entire follow-up period, whereas the incidence did not differ between participants with a persistent healthy weight and those with midlife weight loss (Figure 2). At the end of the HBS follow-up, the median (IQR) age of survivors in HBS was 91 (89-93) years and a similar pattern was observed for mortality. Mean (SD) survival times (1974-2021) were 33.9 (12.0) years, 33.0 (12.5) years, 32.3 (11.9) years, and 31.0 (12.0) years in the persistent healthy weight, weight loss, weight gain, and persistent overweight groups, respectively (*P* < .001).

During a median (IQR) follow-up of 22.8 (16.9-23.3) years, after adjustment for smoking history, systolic blood pressure, and serum cholesterol at the first evaluation, WHII participants with persistent healthy weight (HR, 0.60; 95% CI, 0.54-0.68), weight loss (HR, 0.52; 95% CI, 0.35-0.78), and weight gain (HR, 0.77; 95% CI, 0.65-0.90) were at a decreased risk of first chronic disease compared with those with persistent overweight (Table 2). The decreased risk associated with weight loss remained after excluding diabetes from the outcome (HR, 0.58; 95% CI, 0.37-0.90). The pattern of results was similar for mortality risk in the HBS (eg, weight loss: HR, 0.81; 95% CI, 0.68-0.96) with a median follow-up 35 (23-43) years, and the associations were not materially different among 1743 HBS participants without chronic medications or diseases in the second evaluation (eTable 4 in Supplement 1) or after additional adjustment for change in BMI during follow-up (eTable 5 in Supplement 1).

**Table 2.** Association of Weight Change With Incident Chronic Disease and Death

Weight group^a^	Participants, No. with outcomes/total No. (%)	HR (95% CI)		
		Model 1^b^	Model 2^c^	Model 3^d^
**Primary analysis**				
Outcome: first chronic disease (Whitehall II)				
Persistent healthy weight	634/2161 (29.3)	0.56 (0.50-0.62)	0.57 (0.51-0.64)	0.60 (0.54-0.68)
Weight loss	26/96 (27.1)	0.52 (0.35-0.76)	0.53 (0.36-0.78)	0.52 (0.35-0.78)
Weight gain	204/565 (36.1)	0.73 (0.62-0.85)	0.74 (0.63-0.86)	0.77 (0.65-0.90)
Persistent overweight	586/1296 (45.2)	1 [Reference]	1 [Reference]	1 [Reference]
Outcome: first chronic disease except diabetes (Whitehall II)				
Persistent healthy weight	540/2161 (25)	0.67 (0.59-0.76)	0.69 (0.61-0.78)	0.72 (0.64-0.82)
Weight loss	21/96 (21.9)	0.58 (0.38-0.90)	0.60 (0.39-0.93)	0.58 (0.37-0.90)
Weight gain	167/565 (29.6)	0.82 (0.69-0.98)	0.83 (0.70-0.99)	0.86 (0.71-1.02)
Persistent overweight	445/1296 (34.3)	1 [Reference]	1 [Reference]	1 [Reference]
Outcome: death (HBS)				
Persistent healthy weight	614/750 (81.9)	0.76 (0.69-0.84)	0.77 (0.70-0.85)	0.84 (0.76-0.93)
Weight loss	153/188 (81.4)	0.80 (0.68-0.95)	0.78 (0.66-0.93)	0.81 (0.68-0.96)
Weight gain	143/170 (84.1)	0.88 (0.74-1.05)	0.90 (0.76-1.08)	0.99 (0.83-1.18)
Persistent overweight	1095/1227 (89.2)	1 [Reference]	1 [Reference]	1 [Reference]
**Replication analysis (FPS)**				
Outcome: first chronic disease				
Persistent healthy weight	616/8618 (7.1)	0.38 (0.35-0.42)	0.40 (0.36-0.44)	0.42 (0.38-0.47)
Weight loss	23/332 (6.9)	0.43 (0.28-0.65)	0.43 (0.28-0.65)	0.43 (0.29-0.66)
Weight gain	174/1847 (9.4)	0.58 (0.49-0.68)	0.60 (0.51-0.70)	0.61 (0.52-0.71)
Persistent overweight	1035/5899 (17.5)	1 [Reference]	1 [Reference]	1 [Reference]
Outcome: first chronic disease except diabetes				
Persistent healthy weight	570/8618 (6.6)	0.65 (0.58-0.73)	0.67 (0.60-0.75)	0.70 (0.62-0.79)
Weight loss	16/332 (4.8)	0.54 (0.33-0.89)	0.54 (0.33-0.89)	0.55 (0.33-0.90)
Weight gain	142/1847 (7.7)	0.86 (0.72-1.04)	0.89 (0.74-1.07)	0.90 (0.75-1.08)
Persistent overweight	583/5899 (9.9)	1 [Reference]	1 [Reference]	1 [Reference]

Abbreviations: FPS, Finnish Public Sector study; HBS, Helsinki Businessmen Study; HR, hazard ratio.

^a^ Healthy weight was defined as a body mass index (calculated as weight in kilograms divided by height in meters squared) less than 25, and overweight was defined as a BMI of 25 or greater.

^b^ Unadjusted.

^c^ Adjusted for age and sex.

^d^ Adjusted for age, sex, smoking history, systolic blood pressure, and total cholesterol at the first evaluation.

We repeated these analyses in the independent FPS cohort of participants aged 18 to 48 years (eTables 6 and 7 in Supplement 1). During a median (IQR) follow-up of 12.2 (8.2-12.2) years, after adjustment for age, sex, smoking history, and hypertension, the pattern of results in the primary analysis was replicated. The HR for participants with weight loss compared with those with persistent overweight was 0.43 (95% CI, 0.29-0.66) before and 0.70 (95% CI, 0.62-0.79) after excluding diabetes from the incident chronic disease outcome (Table 2). This finding was little changed after additional adjustment for change in BMI during the follow-up (eTable 8 in Supplement 1). Mortality follow-up of the FPS revealed 181 deaths, with a mean (SD) age at death of 54.2 (5.2) years, including fewer than 5 deaths in the weight loss group.

In sex-specific analyses, including 2968 WHII men and 13 785 women from the FPS, the risk of chronic diseases in participants with midlife weight loss was decreased compared with those with persistent overweight before and after exclusion of diabetes from the outcome (eTables 9 and 10 in Supplement 1).

## Discussion

This cohort study’s findings, with data from 3 well-characterized prospective cohort studies, support the hypothesis that sustained weight loss from overweight and obesity to a healthy weight during healthy midlife without surgical or pharmacological treatment is associated with long-term health benefits. Compared with persistent overweight, such weight loss was associated with a decreased risk of incident chronic disease, both including and excluding diabetes, over follow-up of 12.2 and 22.8 years, as well as decreased all-cause mortality over a 35-year follow-up.

In previous observational studies and RCTs on behaviorally induced weight reduction,^7,8,9,10,11,12,13,14,15,16,17,18,19,20^ follow-up periods have usually been significantly shorter than in our study. This may have contributed to inconsistencies in findings on major clinical outcomes, such as mortality, which accumulate gradually over time. Furthermore, in observational studies of unselected populations, excluding unintentional weight loss due to disease or frailty is more challenging than in RCTs. Observational studies have often reported an association between weight loss and increased mortality risk,^9,10,16^ likely influenced by older age profiles and preexisting morbidities at baseline. To minimize this bias, we focused on weight loss in relatively young individuals who were disease free. Given this, comparisons with our predominantly healthy and younger cohorts should be drawn primarily from RCTs specifically designed to investigate weight reduction.

The Da Qing Diabetes Prevention Study,^3,21^ Diabetes Prevention Study (DPS),^4^ Diabetes Prevention Program (DPP),^5^ Diabetes Prevention Program Outcomes Study (DPPOS),^26,27^ Finnish National Diabetes Prevention Program (FIN-D2D),^6^ and Look Action for Health in Diabetes (Look AHEAD)^15,19^ trials aimed at intentional weight loss and reported on cardiovascular and mortality outcomes. DPS and DPP demonstrated a decreased diabetes risk in individuals with impaired glucose tolerance; however, no differences in macrovascular complications were observed between intervention and control groups in DPPOS.^26^ Look AHEAD, which focused on participants with type 2 diabetes, showed multiple health benefits but no significant cardiovascular or mortality effects over 10 years.^15,19^ Similarly, FIN-D2D reported a decreased diabetes risk but no cardiovascular benefit.^6^ The absence of cardiovascular and mortality benefits is unexpected given the increased cardiovascular risk among trial participants with glucose intolerance or diabetes. Possible explanations for these null findings include insufficient weight loss compared with surgical or pharmacological treatments; frequent use of preventive medications, such as statins, in control groups; and participants being too old or unwell at study entry.^28^ Additionally, short follow-up durations may have been a critical limiting factor.

The Da Qing study^21^ is the only RCT, to our knowledge, to demonstrate long-term mortality benefits, with cardiovascular effects emerging after 24 years and confirmed at the 30-year follow-up. Our observational findings in European populations, based on extended follow-up periods, align with these results from a Chinese population. Both Da Qing and our study suggest that in healthy individuals with overweight, mortality benefits associated with weight loss take time to become apparent and, while positive, remain modest at best.

In contrast to surgical or pharmacological interventions, where weight loss typically ranges from 20% to 25%,^1,2^ the relative weight reduction of 6.5% observed in our study was more modest. Despite this, our findings highlight clinically meaningful long-term health benefits in the general population. Unlike our participants, individuals undergoing surgical or pharmacological interventions predominantly have obesity and are at higher health risk. It remains uncertain whether the long-term benefits observed in our study extend to these interventions, where greater weight loss may be accompanied by a concurrent and potentially significant loss of lean body mass,^22^ which could pose challenges over time.

Future studies on weight loss should focus on the full life course given that studies have highlighted the importance of early-life BMI, showing that childhood overweight and pubertal weight gain were associated with increased risk of coronary atherosclerosis, although normalization during puberty reversed this risk.^29^ In addition, future studies should cover a wider range of outcomes, including health-related quality of life and the risk of frailty, which is associated with overweight at midlife.^30^

### Strengths and Limitations

The main strengths of this study include the replication of findings across multiple independent cohorts, which minimizes the risk of type I error; extended follow-up periods enabling the evaluation of long-term associations; and assessment of weight loss in a disease-free population before old age, reducing the likelihood of weight loss being driven by underlying disease. However, our study also has several limitations. Given that this is an observational study, we cannot evaluate causality. In addition, no information was available regarding whether weight loss was intentional or unintentional. Weight loss occurred between ages 40 and 50 years, when participants were free (or mostly free, in HBS) of chronic diseases and was associated with retained physical activity levels unlike in all other weight groups, in which physical inactivity increased over time (WHII). Unintentional weight loss vs no weight change may be associated with increased risks of diagnosed disease and all-cause mortality^9,10,16^ and would dilute effect estimates for the weight loss group. This being the case, our findings would represent, if anything, an underestimate of associations with morbidity and mortality. BMI assessment in the first 2 evaluations was based on measured height and weight in WHII and HBS; however, subsequent evaluations in HBS relied on self-reports, while FPS used only self-reported data. These differences may have introduced bias, but this is likely to be small given convergent findings across the 3 cohort studies. The use of predominantly White European study populations limits the generalizability of our findings.

## Conclusions

In this cohort study, findings from 3 prospective cohort studies supported maintaining a healthy weight (BMI <25) throughout life as the best option for overall health. Although correcting midlife overweight without surgical or pharmacological treatment is challenging, our results suggest that it is feasible and may be associated with decreased long-term risk of cardiovascular diseases, other chronic conditions, and mortality outcomes associated with overweight.

## Notes

Supplement 1. **eMethods.** Measurement of weight and height **eTable 1.** Disease criteria for the second clinical evaluation in the Helsinki Businessmen Study **eTable 2.** Number (%) of missing values in primary analysis **eTable 3.** Additional sample characteristics for primary cohorts **eFigure 1.** BMI trajectories by weight group in primary cohorts **eTable 4.** Association of weight group with all-cause death among HBS participants without chronic medications or diseases **eTable 5.** Association of weight change with incident chronic disease and death after adjustment for change in BMI during the follow-up in the primary analysis **eTable 6.** Number (%) of missing values in the replication cohort **eTable 7.** Characteristics of the participants in the replication cohort **eTable 8.** Association of weight change with incident chronic disease and death after adjustment for change in BMI during the follow-up in the replication analysis **eTable 9.** Association of weight change with incident chronic disease in men (Whitehall II study) **eTable 10.** Association of weight change with incident chronic disease in women (FPS) **eAppendix.** Statistical code

Supplement 2.
Data Sharing Statement
