# Supplementary material for: Weight Loss in Midlife, Chronic Disease Incidence, and All-Cause Mortality During Extended Follow-Up
Source: JAMA Netw Open. 2025 May 27;8(5):e2511825. doi: 10.1001/jamanetworkopen.2025.11825 (PMC12117462; doi:10.1001/jamanetworkopen.2025.11825)
Supplement: Supplement 1. — eMethods. Measurement of weight and height eTable 1. Disease criteria for the second clinical evaluation in the Helsinki Businessmen Study eTable 2. Number (%) of missing values in primary analysis eTable 3. Additional sample characteristics for primary cohorts eFigure 1. BMI trajectories by weight group in primary cohorts eTable 4. Association of weight group with all-cause death among HBS participants without chronic medications or diseases eTable 5. Association of weight change with incident chronic disease and death after adjustment for change in BMI during the follow-up in the primary analysis eTable 6. Number (%) of missing values in the replication cohort eTable 7. Characteristics of the participants in the replication cohort eTable 8. Association of weight change with incident chronic disease and death after adjustment for change in BMI during the follow-up in the replication analysis eTable 9. Association of weight change with incident chronic disease in men (Whitehall II study) eTable 10. Association of weight change with incident chronic disease in women (FPS) eAppendix. Statistical code [file jamanetwopen-e2511825-s001.pdf]

Supplemental Online Content

Strandberg TE, Strandberg AY, Jyväkorpi S, et al. Weight loss in midlife, chronic disease incidence, and all-cause mortality during extended follow-up. *JAMA Netw Open*. 2025;8(5):e2511825. doi:10.1001/jamanetworkopen.2025.11825

eMethods. Measurement of weight and height

eTable 1. Disease criteria for the second clinical evaluation in the Helsinki Businessmen Study

eTable 2. Number (%) of missing values in primary analysis

eTable 3. Additional sample characteristics for primary cohorts

eFigure 1. BMI trajectories by weight group in primary cohorts

eTable 4. Association of weight group with all-cause death among HBS participants without chronic medications or diseases

eTable 5. Association of weight change with incident chronic disease and death after adjustment for change in BMI during the follow-up in the primary analysis

eTable 6. Number (%) of missing values in the replication cohort

eTable 7. Characteristics of the participants in the replication cohort

eTable 8. Association of weight change with incident chronic disease and death after adjustment for change in BMI during the follow-up in the replication analysis

eTable 9. Association of weight change with incident chronic disease in men (Whitehall II study)

eTable 10. Association of weight change with incident chronic disease in women (FPS)

eAppendix. Statistical code

This supplemental material has been provided by the authors to give readers additional information about their work.

© 2025 Strandberg TE et al. *JAMA Network Open*.

eMethods. Measurement of weight and height

In the 1st and 2nd WHII evaluations, the participants wore only underwear for their weight measurement, which was to the nearest 0.1 kg on Soehnle electronic scales. For height measurements, to the nearest 1 mm using a stadiometer, the participants had bare feet and stood erect with their heads in the Frankfort plane. The repeatability of the weight and height measurements over one month (that is, between participant variability/total [between+within participant] variability), taken from 306 participants, was 0.99. After the first and second examination (1985-1988 and 1991-1993), assessment of BMI was repeated four times (1997–1999, 2002–2004, 2007–2009 and 2012–2013).

In the HBS, height and weight were directly measured at 1st and 2nd evaluation, but self-reported in later follow-ups in 2000, 2002/2003, 2007, and 2010/2011. In 2002/03 a random subgroup of 472 participants was clinically examined, and we found a strong correlation between reported and measured weight (Spearman correlation r = 0.93, P< 0.001). In FPS, both height and weight were self-reported.

We defined healthy weight as BMI less than 25 kg/m^2 and overweight as BMI $\geq$25 kg/m^2. Using weight and height measurements from the 1st and 2nd evaluation, we categorized participants into four groups: 1) Healthy BMI at both evaluations, 2) Overweight at the 1st evaluation and healthy BMI at the 2nd, 3) Healthy BMI at the 1st evaluation and overweight at the 2nd, and 4) Overweight at both evaluations.

In FPS, a larger sample size allowed a definition of weight groups based on three consecutive surveys on self-reported weight and height from surveys in 2000–2002, 2004–2005, 2008–2009 and 2011–2013: persistent healthy weight (healthy BMI in all three consecutive surveys), sustained weight loss (overweight in the first survey, healthy BMI in two subsequent surveys), weight gain (healthy BMI in the first survey, overweight in two subsequent surveys), and persistent overweight (overweight in all three surveys).

© 2025 Strandberg TE et al. *JAMA Network Open*.

eTable 1. Disease criteria for the 2nd clinical evaluation in the Helsinki Businessmen Study

1. Treated, secondary or severe hypertension (blood pressure ≥200 mmHg systolic or ≥ 115 mmHg diastolic
2. Cardiovascular diseases:
- history of myocardial infarction or
- typical angina pectoris
- defined ECG findings related to coronary artery disease, arrhythmias or conduction defects
- cardiomyopathies
- heart valve diseases
- heart failure
3. Cerebrovascular diseases
- history of stroke
- neurologic defects
4. Renal diseases
5. Metabolic diseases
   - medical treatment for diabetes
   -fasting blood glucose ≥10 mmol/L
6. Cancer
7. Psychiatric diseases
   - psychoses
   - alcoholism

© 2025 Strandberg TE et al. *JAMA Network Open*.

eTable 2. Number (%) of missing values in primary analysis

	Whitehall	HBS
**1st Evaluation**		
Age	0 (0.0)	0 (0.0)
BMI	0 (0.0)	0 (0.0)
BMI at age 25	146 (3.5)	162 (6.9)
Height in men	0 (0.0)	0 (0.0)
Height in women	0 (0.0)	0 (0.0)
Smoker	36 (0.9)	0 (0.0)
Systolic blood pressure	3 (0.1)	10 (0.4)
Diastolic blood pressure	3 (0.1)	10 (0.4)
Total cholesterol	20 (0.5)	8 (0.3)
**2nd Evaluation**		
Age	0 (0.0)	0 (0.0)
BMI	0 (0.0)	0 (0.0)

BMI, body mass index, HBS, Helsinki Businessmen study

© 2025 Strandberg TE et al. *JAMA Network Open*.

eTable 3. Additional sample characteristics for primary cohorts

		Whitehall II study						Helsinki Businessmen study					
Characteristic	All (n=4118)	Persistent healthy weight (n = 2161)	Weight loss (n = 96)	Weight gain (n = 565)	Persistent overweight (n = 1296)	Pvalue		All (n=2335)	Persistent healthy weight (n = 750)	Weight loss (n = 188)	Weight gain (n = 170)	Persistent overweight (n = 1227)	P-value
Mean (SD) systolic BP, 2nd evaluation, mmHg	118.8 (12.7)	116.3 (12.4)	117.5 (14.3)	118.9 (11.2)	123.2 (12.6)	<0.001		143 (18.9)	139 (18.7)	141 (20.6)	140 (17.8)	146 (18.3)	<0.001
Mean (SD) diastolic BP, 2nd evaluation, mmHg	79.1 (9.3)	76.7 (8.8)	76.6 (10.0)	80.2 (8.2)	82.8 (9.1)	<0.001		92 (11.3)	88 (10.9)	89 (11.0)	91 (10.9)	95 (11.0)	<0.001
Mean (SD) serum total cholesterol, 2nd evaluation, mg/dL	243.4 (43.5)	236.5 (41.1)	231.3 (41.1)	251.2 (44.2)	252.3 (45.0)	<0.001		243 (41.1)	237 (39.2)	237 (39.2)	251 (42.8)	246 (41.7)	<0.001
Mean (SD) 1-hr glucose at 1st evaluation, mg/dL								114 (33.5)	106 (28.4)	117 (36.0)	110 (27.1)	118 (35.8)	<0.001
Post-load glucose 162 mgl/dL or higher[a], %								20 (8.8)	32 (4.3)	20 (10.4)	7 (4.2)	148 (12.1)	<0.001
Mean (SD) 1-hr glucose at 2nd evaluation, mg/dL								129 (39.1)	120 (33.3)	123 (38.8)	128 (35.2)	135 (41.6)	<0.001
Post-load glucose 162 mg/dL or higher[a], (%)								429 (18.4)	86 (11.4)	27 (14.6)	25 (14.7)	279 (22.7)	<0.001
Mean (SD) 2-hr glucose at 2nd evaluation, mg/dL	97.5 (30.7)	94.9 (28.0)	93.4 (27.5)	96.8 (29.1)	102.5 (35.0)	<0.001							
Post-load glucose 141 mg/dL or higher[b], No. (%)	300 (7.7)	124 (6.0)	5 (5.4)	41 (7.8)	130 (10.7)	<0.001							
Low physical activity, 1st evaluation, No. (%)	477 (11.7)	217 (10.1)	15 (15.8)	55 (9.9)	190 (14.9)	<0.001							
Low physical activity, 2nd evaluation, No. (%)	660 (16.4)	296 (13.9)	15 (16.0)	86 (15.9)	263 (21.0)	<0.001							

[a] Threshold of prediabetes for 1-hour postload glucose.

[b] Threshold of prediabetes for 2-hour postload glucose.

To convert serum total cholesterol from mg/dL to mmol/l, multiply by 0.0258. To convert serum glucose from mg/dL to mmol/l, multiply by 0.0555.

© 2025 Strandberg TE et al. *JAMA Network Open*.

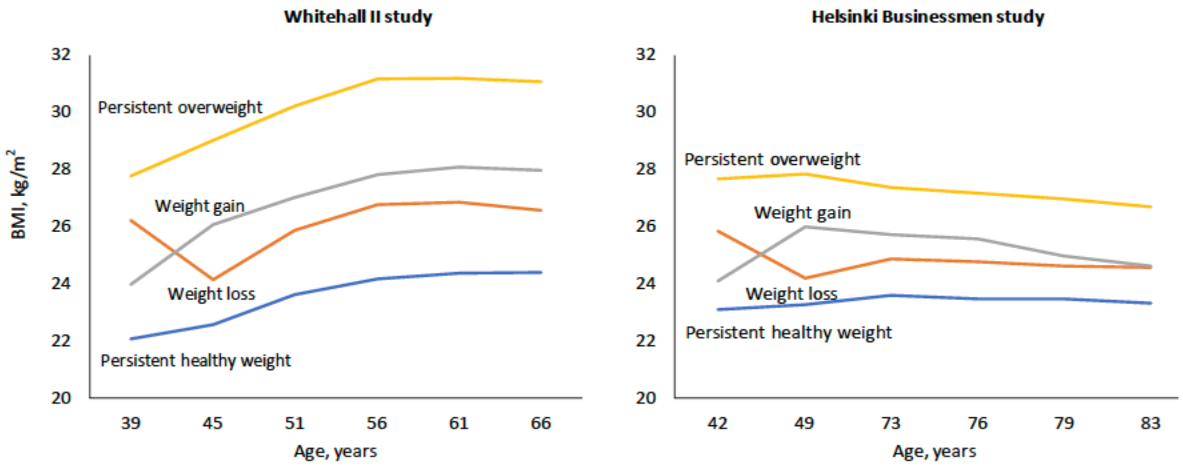

eFigure 1. BMI trajectories by weight group in primary cohorts

© 2025 Strandberg TE et al. *JAMA Network Open*.

eTable 4. Association of weight group with all-cause death among participants (n=1743) of the Helsinki Businessmen Study without chronic medications or diseases listed in eTable 1.

Weight group	N (incident cases/deaths)	Hazard ratio (95% confidence interval)		
		Model 1[a]	Model 2[b]	Model 3[c]
		Outcome: Death (Helsinki Businessmen study)		
Persistent healthy weight	592 (474)	0.77 (0.69-0.87)	0.79 (0.70-0.88)	0.83 (0.74-0.94)
Weight loss	127 (99)	0.78 (0.63-0.96)	0.76 (0.61-0.93)	0.79 (0.63-0.97)
Weight gain	129 (106)	0.92 (0.75-1.13)	0.97 (0.79-1.18)	1.04 (0.84-1.27)
Persistent overweight	895 (785)	1.00 (reference)	1.00 (reference)	1.00 (reference)

[a] Unadjusted
[b] Adjusted for age
[c] Adjusted for age, smoking, systolic blood pressure and total cholesterol at 1st evaluation

eTable 5. Association of weight change with incident chronic disease and death after adjustment for change in BMI during the follow-up in primary analysis

Weight group	Total No. (Cases No.)	Hazard ratio (95% confidence interval)[a]
		Outcome: First chronic disease (Whitehall)
Persistent healthy weight	2129 (627)	0.61 (0.54-0.69)
Weight loss	94 (25)	0.52 (0.35-0.77)
Weight gain	559 (203)	0.77 (0.66-0.91)
Persistent overweight	1278 (577)	1.00 (reference)
		Outcome: First chronic disease except diabetes (Whitehall)
Persistent healthy weight	2129 (535)	0.72 (0.63-0.82)
Weight loss	94 (20)	0.58 (0.37-0.91)
Weight gain	559 (166)	0.85 (0.71-1.02)
Persistent overweight	1278 (438)	1.00 (reference)
		Outcome: Death (Helsinki Businessmen study)
Persistent healthy weight	747 (611)	0.87 (0.79-0.97)
Weight loss	184 (150)	0.85 (0.71-1.01)
Weight gain	169 (142)	0.99 (0.83-1.18)
Persistent overweight	1217 (1085)	1.00 (reference)

[a] Adjusted for age, sex, smoking, systolic blood pressure and BMI change during follow-up

eTable 6. Number (%) of missing values in the replication cohort (Finnish Public Sector study)

	FPS
**1st Evaluation**	
Age	0 (0.0)
BMI	0 (0.0)
BMI at age 25	–
Height in men	0 (0.0)
Height in women	0 (0.0)
Smoker	4 (0.02)
Hypertension	0 (0.0)
**2nd Evaluation**	
Age	0 (0.0)
BMI	0 (0.0)

BMI, body mass index, FPS, Finnish Public Sector study.

© 2025 Strandberg TE et al. *JAMA Network Open*.

eTable 7. Characteristics of the participants in the replication cohort (Finnish Public Sector study)

	Weight change between 1st and 2nd evaluation[a] (FPS)					
Characteristic	All (n=16696)	Persistent healthy weight (n = 8618)	Weight loss (n = 332)	Weight gain (n = 1847)	Persistent overweight (n = 5899)	P-value
**1st Evaluation**						
Proportion of men, No. (%)	2911 (17.4)	979 (11.4)	71 (21.4)	337 (18.3)	1524 (25.8)	<0.001
Age, median (IQR), y	39 (34–43)	39 (33–43)	40 (36–44)	39 (34–43)	40 (35–44)	<0.001
Mean (SD) BMI, kg/m^2	24.5 (4.1)	21.6 (1.6)	26.1 (1.7)	23.9 (1.1)	28.8 (3.5)	<0.001
Obesity, No. (%)	1627 (9.7)	0 (0.0)	8 (2.4)	0 (0.0)	1619 (27.5)	<0.001
Mean (SD) height in men, cm	180 (6.2)	180 (6.2)	180 (6.1)	180 (6.4)	180 (6.3)	0.66
Mean (SD) height in women, cm	166 (5.8)	166 (5.7)	166 (6.1)	166 (5.8)	165 (5.8)	<0.001
Current smoker, No. (%)	2943 (17.6)	1238 (14.4)	69 (20.8)	375 (20.3)	1261 (21.4)	<0.001
Ex-smoker, No. (%)	2920 (17.5)	1398 (16.2)	58 (17.5)	303 (16.4)	1161 (19.7)	<0.001
Current or ex-smoker, No. (%)	5863 (35.1)	2636 (30.6)	127 (38.3)	678 (36.7)	2422 (41.1)	<0.001
Hypertension, No. (%)	452 (2.7)	115 (1.3)	11 (3.3)	50 (2.7)	276 (4.7)	<0.001
**2nd Evaluation**						
Age, median (IQR), y	43 (38–47)	42 (37–47)	44 (40–47)	43 (38–47)	44 (39–47)	<0.001
Mean (SD) years since 1st evaluation	3.7 (0.7)	3.7 (0.8)	3.9 (0.7)	3.9 (0.7)	3.7 (0.7)	<0.001
Mean (SD) BMI, kg/m^2	25.2 (4.4)	21.9 (1.6)	23.5 (1.2)	26.4 (1.6)	29.6 (3.7)	<0.001
Obesity, No. (%)	2160 (12.9)	0 (0.0)	0 (0.0)	42 (2.3)	2118 (35.9)	<0.001
Mean (SD) weight change, kg	1.9 (5.1)	0.9 (2.9)	-7.3 (7.0)	6.9 (5.4)	2.3 (6.0)	<0.001
Relative (SD) weight change, %	2.9 (7.0)	1.6 (4.9)	-9.5 (7.1)	10.5 (9.2)	3.0 (6.8)	<0.001

[a]The first and second evaluations were in 2000-2002 and 2004-2005 in the Finnish Public Sector study (FPS).
Abbreviations. BMI, body mass index; IQR, interquartile range; SE, standard error; y, year.

eTable 8. Association of weight change with incident chronic disease and death after adjustment for change in BMI during the follow-up in the replication analysis

Weight group	Total No. (Cases No.)	Hazard ratio (95% confidence interval)[a]
		Outcome: First chronic disease (FPS)
Persistent healthy weight	8618 (616)	0.44 (0.39-0.48)
Weight loss	332 (23)	0.45 (0.30-0.68)
Weight gain	1847 (174)	0.61 (0.52-0.72)
Persistent overweight	5899 (1035)	1.00 (reference)
		Outcome: First chronic disease except diabetes (FPS)
Persistent healthy weight	8618 (570)	0.71 (0.63-0.79)
Weight loss	332 (16)	0.55 (0.34-0.91)
Weight gain	1847 (142)	0.90 (0.75-1.08)
Persistent overweight	5899 (583)	1.00 (reference)

[a] Adjusted for age, sex, smoking, systolic blood pressure and BMI change during follow-up

© 2025 Strandberg TE et al. *JAMA Network Open*.

eTable 9. Association of weight change with incident chronic disease in men (Whitehall II study)

Weight group	No. Total	No. Cases	Hazard ratio (95% confidence interval)[a]
			Outcome: First chronic disease
Persistent healthy weight	1538	458	0.65 (0.56-0.74)
Weight loss	72	20	0.57 (0.36-0.89)
Weight gain	405	153	0.86 (0.71-1.04)
Persistent overweight	953	421	1.00 (reference)
			Outcome: First chronic disease except diabetes
Persistent healthy weight	1538	383	0.75 (0.64-0.87)
Weight loss	72	16	0.61 (0.37-1.01)
Weight gain	405	124	0.94 (0.76-1.16)
Persistent overweight	953	321	1.00 (reference)

[a]Adjusted for age, sex, smoking, and systolic blood pressure at first evaluation

eTable 10. Association of weight change with incident chronic disease in women (Finnish Public Sector study)

			Hazard ratio (95% confidence interval)[a]		
**Weight group**	**N(total)**	**N(cases)**	**All**	**Women aged < 50 y**	**Women aged 50+ y**
			**Outcome: First chronic disease**		
Persistent healthy weight	7639	559	0.42 (0.38-0.47)	0.45 (0.38-0.53)	0.40 (0.34-0.46)
Weight loss	261	18	0.42 (0.26-0.66)	0.48 (0.25-0.93)	0.37 (0.19-0.71)
Weight gain	1510	135	0.55 (0.46-0.66)	0.52 (0.39-0.68)	0.58 (0.46-0.74)
Persistent overweight	4375	800	1.00 (reference)	1.00 (reference)	1.00 (reference)
			**Outcome: First chronic disease except diabetes**		
Persistent healthy weight	7639	519	0.69 (0.61-0.78)	0.72 (0.60-0.87)	0.67 (0.56-0.79)
Weight loss	261	14	0.58 (0.34-0.98)	0.63 (0.30-1.33)	0.53 (0.25-1.13)
Weight gain	1510	115	0.84 (0.68-1.03)	0.75 (0.54-1.02)	0.91 (0.70-1.20)
Persistent overweight	4375	464	1.00 (reference)	1.00 (reference)	1.00 (reference)

[a]Adjusted for age, sex, smoking, and systolic blood pressure at first evaluation

© 2025 Strandberg TE et al. *JAMA Network Open*.

eAppendix
Statistical code (SAS, version 9.4):

```
*********************************************************************;
***  Weight change vs. incident chronic disease  ***;
*********************************************************************;
proc freq data=b1;
     tables bmic*(status_dis status_nondb status_mort) / nopercent nocol;
run;
proc phreg data=b1;
     class bmic(ref='4');
     model ftime_dis*status_dis(0) = bmic / rl;
*    model ftime_nondb*status_nondb(0) = bmic / rl;
*    model ftime_mort*status_mort(0) = bmic / rl;
run;
proc phreg data=b1;
     class bmic(ref='4');
     model ftime_dis*status_dis(0) = sex age bmic / rl;
*    model ftime_nondb*status_nondb(0) = sex age bmic / rl;
*    model ftime_mort*status_mort(0) = sex age bmic / rl;
run;
proc phreg data=b1;
     class smoke bmic(ref='4');
     model ftime_dis*status_dis(0) = sex age hypert smoke bmic / rl;
*    model ftime_nondb*status_nondb(0) = sex age hypert smoke bmic / rl;
*    model ftime_mort*status_mort(0) = sex age hypert smoke bmic / rl;
run;

*********************************************************************;
***  Cumulative incidence of chronic disease  ***;
*********************************************************************;
proc phreg data=b1;
     model ftime_dis*status_dis(0) = ;
     strata bmic;
     baseline out=apu1 survival=survival;
run;
data apu2;
     set apu1;
     rename ftime_dis=time bmic=group;
run;
data apu3;
     set apu2;
     by group;
     retain prehazard 0;
     hazard=1-survival;
     if (hazard>. and hazard NE prehazard) or first.group;
     prehazard=hazard;
     keep group time hazard;
data apu4;
     set apu3;
     retain prehazard pregroup 0;
     if group=pregroup then do;
        cumhazard=prehazard; output; end;
     cumhazard=hazard; output;
     pregroup=group;
     prehazard=hazard;
     keep group time cumhazard;
data apu4;
     set apu4;
     cumhazard=100*cumhazard; ** % **;
run;
proc print data=apu4; ** => Excel figure **;
run;
** Number at risk **;
data n1;
     set b1;
     f0n=0; f4n=0; f8n=0; f12n=0;
     if ftime_dis>=0 then f0n=1;
     if ftime_dis>=4 then f4n=1;
     if ftime_dis>=8 then f8n=1;
     if ftime_dis>=12 then f12n=1;
run;
proc means data=n1 sum;
     var f0n--f12n;
     class bmic;
run;

*********************************************************************;
***  Adjustment for change in BMI during the follow-up  ***;
*********************************************************************;
data b2;
     set b1;
     if bmi3>. then do; bmichange3=bmi3-bmi2; time3=age3-age2; end;
     if bmi4>. then do; bmichange4=bmi4-bmi2; time4=age4-age2; end;
     if bmi5>. then do; bmichange5=bmi5-bmi2; time5=age5-age2; end;
run;
proc phreg data=b2;
     class smoke bmic(ref='4');
     model ftime_dis*status_dis(0)= sex age smoke hypert timedep_bmi bmic / rl;
     timedep_bmi=0;
     if time3>0 and ftime_dis>=time3 then timedep_bmi=bmichange3;
```

© 2025 Strandberg TE et al. *JAMA Network Open*.

```
     if time4>0 and ftime_dis>=time4 then timedep_bmi=bmichange4;
     if time5>0 and ftime_dis>=time5 then timedep_bmi=bmichange5;
run;

********************************************************************;
***  Weight change vs. incident chronic disease / WOMEN  ***;
********************************************************************;
data women;
     set b1;
     IF sex=2;
     time50=50-age2;

     *women aged <50 years*;
/*   IF ftime_dis>time50 then do; ftime_dis=time50; status_dis=0; end;  */
/*   IF ftime_nondb>time50 then do; ftime_nondb=time50; status_nondb=0; end; */

     *women aged 50+ years*;
/*   IF ftime_dis>=time50; ftime_dis= ftime_dis-time50; age=50; */
/*   IF ftime_nondb>=time50; ftime_nondb= ftime_nondb-time50; age=50; */
run;
proc freq data=women;
     tables bmic* status_dis/ nopercent nocol;
*    tables bmic* status_nondb/ nopercent nocol;
run;
proc phreg data=women;
     class bmic(ref='4');
     model ftime_dis* status_dis (0) = sex age hypert smoke bmic / rl;
*    model ftime_nondb* status_nondb (0) = sex age hypert smoke bmic / rl;
run;
```

© 2025 Strandberg TE et al. *JAMA Network Open*.
