# Supplementary material for: Weight Loss in Midlife, Chronic Disease Incidence, and All-Cause Mortality During Extended Follow-Up
Source: JAMA Netw Open. 2025 May 27;8(5):e2511825. doi: 10.1001/jamanetworkopen.2025.11825 (PMC12117462; doi:10.1001/jamanetworkopen.2025.11825)
Supplement: Supplement 2. — Data Sharing Statement [file jamanetwopen-e2511825-s002.pdf]

Data Sharing Statement

Strandberg. Weight Loss in Midlife, Chronic Disease Incidence, and All-Cause Mortality During Extended Follow-Up. *JAMA Netw Open*. Published online May 27, 2025. doi:10.1001/jamanetworkopen.2025.11825

Data

Data available: Yes
Data types: Deidentified participant data
How to access data: Data, protocols, and other metadata for the Whitehall II study are available to the scientific community. Please refer to the Whitehall II study data sharing policy at: https://www.ucl.ac.uk/whitehallII/data-sharing . Data on HSB and FPS are stored in a protected server environment hosted by University of Helsinki and Finnish Institute of Occupational Health, Finland. Pseudonymised questionnaire data can be requested from the principal investigator by contacting Dr Timo Strandberg (timo.strandberg@helsinki.fi), HBS, and Dr Jenni Ervasti (jenni.ervasti@ttl.fi), FPS. Linked health records for HBS and FPS additionally require separate permission from the Findata, the Health and Social Data Permit Authority.
When available: With publication

Supporting Documents

Document types: None

Additional Information

Who can access the data: researchers whose proposed use of the data has been approved
Types of analyses: specified purpose
Mechanisms of data availability: with investigator support
Any additional restrictions: None
